# Use of PEG-asparaginase in a case of Hepatosplenic γδ T-cell lymphoma with long-term remission after stem cell transplantation

**DOI:** 10.3332/ecancer.2018.872

**Published:** 2018-09-20

**Authors:** Kai Sun, Cesar Gentille Sanchez, Sai Ravi Pingali, Swaminathan Iyer

**Affiliations:** 1 Department of Medicine, Houston Methodist Hospital, Houston, TX 77030, USA; 2 Department of Hematology, Houston Methodist Hospital, Houston, TX 77030, USA

**Keywords:** Hepatosplenic γδ T-cell lymphoma, pegylated-asparaginase, stem cell transplant

## Abstract

Hepatosplenic γδ T-cell lymphoma (HSTCL) is a rare aggressive peripheral T-cell lymphoma. Prognosis is usually poor with a median survival between 8 and 16 months after traditional chemotherapy. Stem cell transplantation (SCT) is promising and with a more intense induction regimen, has yielded positive results. We report the use of pegylated-asparaginase (PEG-asparaginase) along with a conventional anthracycline-containing regimen in a 51-year-old male who was diagnosed with HSTCL, achieved a complete remission, and subsequently underwent peripheral blood SCT and remained in remission at the time of this case report.

## Background

Hepatosplenic γδ T-cell lymphoma (HSTCL) is a rare peripheral T-cell lymphoma that comprises 3% of all T-cell lymphoma subtypes in the United States, 2.3% in Europe and 0.2% in Asia [1]. It is resistant to conventional chemotherapy with a median survival time of between 8 and 16 months [2–4]. Since its first description in 1990 [5], no significant breakthrough in treatment has occurred despite ongoing experiments with new therapeutic drugs and stem cell transplantation (SCT). We report a case of HSTCL treated with conventional chemotherapy with PEG-asparaginase, followed by peripheral blood SCT, who achieved remission for a period of more than 52 months.

## Case presentation

A 51-year-old Bolivian male with a past medical history of long-standing rheumatoid arthritis which was treated with methotrexate and steroids 10 years prior to his presentation when he noticed an enlarged spleen. He was diagnosed with Felty’s syndrome and was treated with corticosteroids. Later, he was found to have a decreased white cell count which was treated with filgrastim. Several months before hospitalisation, the patient experienced increasing fatigue, night sweats and weight loss of 10 Ibs and was admitted to the hospital after developing nausea. There was no history of fever, joint pain or skin rash.

On physical examination, vital signs included a blood pressure of 126/77 mmHg, heart rate of 93 bpm, respiratory rate of 12/minutes and temperature of 36.5 °C. Abdominal examination revealed a flat abdomen with a total liver span of 10 cm and a splenomegaly of 15–16 cm below the left costal margin. There was no lymphadenopathy in the cervical, supraclavicular or axillary areas. Laboratory findings were significant for leukopenia (white blood cell 0.58 × 10^9^/L), anaemia (Hgb 8.5 g/dL) and thrombocytopenia (platelets 54 × 10^9^/L), i.e., a pancytopenia.

A computerised tomographic scan of the chest, abdomen and pelvis showed a massively enlarged spleen measuring 12.3 × 21.2 × 30.1 cm. There was a poorly defined mass in the spleen consistent with lymphomatous involvement. The liver was also enlarged measuring 18.2 × 20.2 × 21.5 cm. Bone marrow biopsy revealed small to intermediate sized T lymphocytes in an interstitial and intrasinusoidal distribution involving approximately 30% of the cellularity of a 98% cellular marrow and mild reticulin fibrosis; flow cytometry revealed T lymphocytes that were positive for CD3, CD7, CD2, CD45 and TCR-γδ and negative for CD5, CD4, CD8 and TCR-αβ. Chromosomal abnormalities were not detected. Based on these findings, a diagnosis of HSTCL was made.

## Treatment

The patient received five cycles of EPOCH (etoposide, prednisone, vincristine, cyclophosphamide and doxorubicin) with PEG-asparaginase on the sixth day of the last three cycles. Complete remission (CR) was confirmed by bone marrow biopsy, flow cytometry and positron emission tomography (PET) scan after the fourth cycle. A computerised tomographic scan of the chest, abdomen and pelvis after the last cycle showed a significant decrease in the size of the spleen measuring 9.2 cm × 16 cm × 21.7 cm while the liver size didn’t change much measuring 19.6 cm × 19.2 cm × 20.4 cm. Two months after the last cycle, he received cyclophosphamide and total body irradiation as conditioning for SCT and was transplanted with peripheral blood stem cells from a matched related donor (10/10 match). He engrafted on post-transplantation day 18. Hospitalisation after transplantation was complicated by *E. coli sepsis* which was successfully treated with antibiotics. He received tacrolimus and methotrexate for graft-versus-host disease (GVHD) prophylaxis in the hospital and was discharged on tacrolimus and prednisone.

## Follow-up

The patient discontinued his tacrolimus 13 months after transplantation and returned with chronic skin GVHD with sclerodermatous changes. He received two cycles of rituximab with some improvement of the skin changes, but it was not possible to taper the prednisone further. The patient was started on ruxolitinib and tacrolimus, and subsequently, the prednisone dose was finally tapered. A bone marrow biopsy at 12 and 48 months after transplantation showed CR with 100% donor chimerism. A PET scan at 12 and 27 months after transplantation showed no evidence of recurrent lymphoma. He has remained in CR for 52 months after transplantation (at the time of submission of this report).

## Discussion

γδ T lymphocytes develop from CD4-/CD8-thymic precursors in the bone marrow and they usually lack the major histocompatibility complex restriction [6, 7]. In conditions like chronic immunosuppression and prolonged antigenic exposure, the uncontrolled growth of γδ T lymphocytes can result in the development of lymphomas, expressing the γδ T-cell receptor (TCR) [8]. Even though the patient’s rheumatoid arthritis was not heavily treated and the treatment was remote, it is still possible that those treatments and the disturbance of his immune system have contributed to the development of his HSTCL. Farcet *et al* [5] first described HSTCL in 1990 as a new entity of peripheral T-cell lymphoma (PTCL). HSTCL is classified as one of the subtypes of mature T-cell/NK-cell lymphoma, according to 2016 WHO classification [9].

Systemic B symptoms (fever of unknown origin, night sweats and weight loss of more than 10% of body weight) along with hepatosplenomegaly and lack of lymphadenopathy are characteristic of the disease [8]. Thrombocytopenia is the most striking finding in almost all the patients and is associated with anaemia and leucopenia in more than 50% of the patients. The bone marrow is involved in about two-thirds of the patients, thus careful histologic and immunophenotypic evaluation of the bone marrow should be adequate for making the diagnosis. Splenectomy is rarely performed for diagnostic purposes nowadays [4].

A common phenotype in HSTCL is CD2+CD3+CD4−CD5−CD7+CD8−TCR-γδ+. NK-related antigens, CD16 and CD56 are frequently expressed. Weidmann reviewed 45 cases of HSTCL, out of which two-thirds expressed CD7, a molecule that acts as an activator of various NK/T-cell populations. In a review of 21 cases by Belhadj *et al* [4], CD56 NK antigen was expressed in 15 out of 18 patients and the authors speculated that the γδ variants of HSTCL could represent proliferation of NK cells. Travert *et al* [10] analysed a series of HSTCL samples in relation to normal γδ cells, peripheral T-cell lymphoma not otherwise specified (PTCL-NOS) and extranodal NK/T-cell lymphoma, nasal type (NKTCL) and revealed that the most overexpressed genes in HSTCL were those associated with NK-cell-associated molecules, such as killer immunoglobulin-like receptors, killer cell lectinlike receptors (KLRs), CD244 and NCAM1. In addition, AIM1, a tumour suppressor gene which was found in NKTCL, was found to have significant down-expression of its mRNA in HSTCL cells. These findings provide more evidence that γδ T cells and NK cells both arise from the innate immune system and might share the same origin [11].

HSTCL is a generally incurable disease. CR is rarely achieved with conventional chemotherapy. The median survival is between 8 and 16 months [2–4]. In the case series reported by Balhadj *et al* [4], 19 out of 21 (90.5%) patients received CHOP (cyclophosphamide, doxorubicin, vincristine and prednisone) or a CHOP-like regimen. 7 of the 19 patients achieved CR or partial remission, which was followed by transplantation. Regardless of transplantation, all 19 patients relapsed with a survival time ranging from 2 to 44 months. The only two patients who were in remission at 42 and 52 months at the time of this report were those who received a platinum-cytarabine-based induction regimen followed by transplantation. In a later review of 15 cases performed by Falchook *et al* [12] in 2009, two out of six patients who were treated with a CHOP regimen achieved CR that lasted 7 and 8 months, respectively. One of the two patients was transplanted but died at 13 months. Four patients received more intense chemotherapy: HyperCVAD/Doxil (fractionated Cytoxan, liposomal doxorubicin, vincristine and dexamethasone) alternating with methotrexate and cytarabine; three achieved CR which was followed by transplantation and they were the only patients who remained in remission afterwards. Another review of 45 cases by Weidmann confirmed the poor response to the CHOP-based regimen [2].

The unsatisfactory response to a CHOP-based regimen prompted further research with other chemotherapeutic drugs. There are several case reports using Pentostatin, a purine analogue and potent adenosine deaminase inhibitor, as a single agent that achieved satisfactory sustained clinical and histologic response [13–17]. The use of a combination of fludarabine with alemtuzumab and the combination of alemtuzumab with cladribine also yielded some positive results in some case reports [18, 19].

SCT is considered a potential cure for HSTCL. There are case reports of long-term overall survival after SCT, especially in pediatric patients [20–22]. In some of the case reviews mentioned earlier, more intense induction regimens were associated with a more significant increase in overall survival compared to a CHOP-based regimen, as induction chemotherapy. Voss *et al* [23] performed a retrospective review of the patients with HSTCL who underwent treatment at Memorial Sloan-Kettering Cancer center and identified 14 patients, 7 out of which remained alive with a median follow-up of 65.6 months. Six of seven received more intense induction chemotherapy regimens such as ICE (ifosfamide, carboplatin and etoposide) or IVAC (ifosfamide, etoposide and high-dose cytarabine) instead of a CHOP-based regimen and all surviving patients received transplantation. Their results again suggested that non-CHOP induction regimens and early use of high dose therapy and SCT may improve the survival of HSTCL patients.

Given the aggressiveness of the disease, we chose EPOCH regimen instead of the CHOP regimen in this patient. PEG-asparaginase was also added. This has rarely been reported in the literature. Asparaginase catalyses the degradation of asparagine, an amino acid that is essential to tumour cell growth [24]. While normal cells contain a high level of asparagine synthetase, tumour cells express low levels of asparagine synthetase and are more susceptible to asparaginase. Asparaginase has been used in acute lymphoblastic leukaemia and non-Hodgkin lymphoma [25, 26]. Conjugation with polyethylene glycol (PEG-asparaginase) preserves asparaginase’s activity and slows the elimination of the drug [27]. In a case report by Schafer *et al* [22], a 17-year-old female with HSTCL was treated with a leukemic-based induction which included PEG-asparaginase, followed by early allogeneic transplantation, achieved CR and remained in remission at 18 months. This demonstrated the benefit potentially brought by PEG-asparaginase.

The mechanism of PEG-asparaginase effectiveness on our HSTCL patient might be similar to that on NK-cell lymphoma. Since Miki Ando *et al* [28] reported the high antitumour activity of L-asparaginase against NK-cell lymphoma cells in vitro, L-asparaginase has been applied to treat stage IV, relapsed or refractory NKTCL and has improved the objective response rate (ORR) to 79% [29, 30]. As mentioned earlier, studies have provided evidence that γδ cells and NK cells are both derived from the innate immune system and might share the same origin [10, 11]. Given the successful use of PEG-asparaginase in NK-cell lymphoma, its use in HSTCL is worth exploring.

## Conclusion

HSTCL is a rare and aggressive T-cell lymphoma. It is usually resistant to conventional chemotherapy. New therapeutic agents have been developed that could potentially treat HSTCL. PEG-asparaginase is a promising new agent given its high antitumour activity and superior response in other related PTCL like NKTCL. Its use as an induction chemotherapeutic drug before transplantation is also worth exploring.

## Conflicts of interest

The author(s) declare that they have no conflicts of interest.

## Funding

There is no funding associated with this case report.
